# Postpartum Depression and Maternal Care: Exploring the Complex Effects on Mothers and Infants

**DOI:** 10.7759/cureus.41381

**Published:** 2023-07-04

**Authors:** Rishika Saharoy, Ashwini Potdukhe, Mayur Wanjari, Avinash B Taksande

**Affiliations:** 1 Medicine, Jawaharlal Nehru Medical College, Datta Meghe Institute of Higher Education and Research, Wardha, IND; 2 Medical Surgical Nursing, Smt. Radhikabai Meghe Memorial College of Nursing, Datta Meghe Institute of Higher Education and Research, Wardha, IND; 3 Research and Development, Jawaharlal Nehru Medical College, Datta Meghe Institute of Higher Education and Research, Wardha, IND; 4 Physiology, Jawaharlal Nehru Medical College, Datta Meghe Institute of Higher Education and Research, Wardha, IND

**Keywords:** interventions, infants, mothers, consequences, maternal care, postpartum depression

## Abstract

Postpartum depression (PPD) is a common and debilitating mental health condition affecting many mothers worldwide. This review article aims to explore the complex effects of PPD on mothers and infants, focusing on maternal care. The transition to motherhood is a critical period characterized by numerous physical, psychological, and social changes, making women vulnerable to the onset of PPD. Consequently, PPD can significantly impact a mother's ability to provide optimal care for her infant, leading to potential adverse consequences for both parties. The article synthesizes existing research literature on the topic, encompassing studies from various disciplines, including psychology, psychiatry, obstetrics, and pediatrics. It begins by providing an overview of the prevalence and risk factors associated with PPD, emphasizing the importance of early detection and intervention. The impact of PPD on maternal caregiving behaviors, such as bonding, sensitivity, and responsiveness, is then examined, highlighting the potential disruptions in the mother-infant relationship. Furthermore, the article delves into the potential consequences of impaired maternal care on infant development, including emotional, cognitive, and social domains. Several factors contributing to the complex interplay between PPD and maternal care are discussed, including hormonal changes, psychosocial stressors, and the influence of social support networks. The review also addresses the bidirectional nature of the mother-infant relationship, whereby infant characteristics and behaviors can exacerbate or mitigate the effects of PPD on maternal care. Moreover, the article explores the role of healthcare providers and the importance of implementing effective screening, assessment, and treatment strategies for PPD to promote optimal maternal-infant outcomes. By consolidating current knowledge on the topic, this review article provides valuable insights into the multifaceted effects of PPD on both mothers and infants. Recognizing the significance of maternal care and understanding the mechanisms through which PPD disrupts it can inform the development of targeted interventions to promote early detection, effective treatment, and supportive interventions for mothers experiencing PPD. Ultimately, improving maternal mental health and enhancing maternal-infant relationships can yield long-term positive effects on mothers' and infants' well-being and development.

## Introduction and background

Postpartum depression (PPD) is a prevalent mental health disorder that affects women after giving birth. It is characterized by persistent sadness, a loss of interest, and a diminished sense of pleasure. PPD is more than just the "baby blues" and can significantly impact a mother's daily functioning and well-being. Approximately 10-20% of mothers are estimated to experience PPD, making it a significant public health concern [1,2]. The postpartum period is a critical time for both mothers and infants. Maternal care during this phase is crucial to both mother's and child's physical, emotional, and psychological well-being. Maternal care encompasses various aspects, such as providing a nurturing and supportive environment, establishing healthy routines, promoting breastfeeding, and addressing the mother's mental health needs [3].

Comprehending the ramifications of PPD on mothers and infants is paramount. In the case of mothers, PPD can result in a diminished quality of life, hindered bonding with the infant, and challenges in fulfilling essential maternal care responsibilities. Moreover, it can adversely affect the mother's mental health and elevate the risk of enduring mental health disorders [4,5]. As for infants, being exposed to a mother experiencing PPD can harm their emotional, cognitive, and social development. They may encounter delays in achieving developmental milestones, exhibit behavioral issues, and face an increased likelihood of developing insecure attachments with their mothers. In the long term, this may heighten the chances of mental health disorders and compromise their overall well-being [6,7].

By gaining insight into the multifaceted consequences of PPD for mothers and infants, healthcare providers, policymakers, and society can implement targeted interventions and support systems to alleviate the negative impacts and foster the well-being of those affected. Further research in this domain can also contribute to developing effective prevention and treatment strategies for PPD and its consequences [8].

This review article aims to comprehensively analyze PPD and its consequences for mothers and infants. By synthesizing existing research, the article aims to deepen our understanding of the impacts of PPD on maternal care and infant outcomes. It seeks to raise awareness about the significance of PPD and contribute to developing effective interventions, support systems, and policy measures to enhance maternal care and promote the well-being of mothers and infants.

## Review

Methodology

The methodology employed for this review article involved conducting a comprehensive literature search to identify relevant studies on PPD and its consequences for mothers and infants. The search strategy encompassed various sources, including scholarly databases, research journals, and reputable websites. Specific search terms related to PPD, maternal care, consequences, mothers, and infants were combined with Boolean operators to refine the search results. Multiple databases, such as PubMed, PsycINFO, Scopus, and Google Scholar, were consulted to ensure a thorough search. In addition, the reference lists of relevant articles and systematic reviews were reviewed for potential inclusion of additional studies. The selection of studies for inclusion followed specific criteria to ensure the relevance and quality of the literature. Only studies published in peer-reviewed journals were considered, ensuring they underwent rigorous review and met scientific standards. The focus was on studies specifically addressing PPD and its consequences for mothers and infants, including the impact on maternal behaviors, maternal-infant attachment, and breastfeeding practices. Studies involving human participants were prioritized, while those focusing on animal models or theoretical frameworks were excluded. The selected studies were published in English and within the last 10 years to include current research. The screening process involved assessing titles, abstracts, and full-text articles for relevance and suitability based on the inclusion criteria. By applying these rigorous inclusion and exclusion criteria, a selection of high-quality studies was identified to provide an evidence-based overview of the consequences of PPD for mothers and infants in this review article.

Prevalence and risk factors of PPD

Statistics on PPD Rates

Statistics on PPD rates indicate that it is a common mental health condition affecting many women worldwide. PPD is estimated to affect around 10-20% of mothers, making it a widespread concern in maternal mental health. These statistics underscore the importance of recognizing and addressing PPD as a significant public health issue [9,10].

The 10-20% range demonstrates the variability in reported rates across different studies and populations. The actual prevalence may vary depending on various factors such as cultural differences, socioeconomic status, access to healthcare, and individual risk factors. However, regardless of the exact prevalence, it is clear that PPD is a relatively common occurrence that can have far-reaching implications for both mothers and infants [11,12]. The high prevalence of PPD highlights the urgent need for increased awareness, screening, and support for women during the postpartum period. Healthcare providers, policymakers, and society as a whole must recognize the prevalence of this condition and ensure that appropriate resources and interventions are in place to address the needs of affected mothers [13].

By increasing awareness and understanding of PPD rates, healthcare providers can be better equipped to identify and support women at risk. Early detection through routine screening can facilitate timely interventions and treatment, reducing the impact of PPD on maternal well-being and infant development [14]. Furthermore, society must work towards reducing the stigma surrounding PPD, encouraging women to seek help without fear of judgment or shame. This involves promoting open discussions, providing accurate information, and creating a supportive environment where affected mothers can receive the care and support they need.

Identification of Risk Factors Associated With PPD

Personal or family history of depression or other mental health disorders: Women with a history of depression or anxiety disorders are more likely to develop PPD. Additionally, having a family history of mental health disorders can increase the susceptibility to PPD. These factors suggest a genetic predisposition and the influence of underlying mental health vulnerabilities [10,15].

Hormonal changes: The significant hormonal fluctuations that occur during the postpartum period, particularly the rapid decline in estrogen and progesterone levels, can contribute to the onset of PPD. These hormonal changes, combined with other factors such as sleep deprivation and stress, can disrupt the delicate balance of neurotransmitters involved in mood regulation, potentially leading to depressive symptoms [16].

Previous experience of PPD: Women who have previously experienced PPD in prior pregnancies have a higher risk of developing it in subsequent pregnancies. The recurrence of PPD indicates the persistence of underlying vulnerabilities or the potential impact of specific situational factors that contribute to its onset [4].

Lack of social support: Adequate social support from partners, family members, and friends is crucial postpartum. The absence or inadequacy of a support network can intensify feelings of isolation, overwhelm, and stress experienced by new mothers, increasing the risk of developing PPD. A strong support system can provide emotional support, practical assistance, and validation of the mother's experiences, which can help alleviate depressive symptoms [17].

Life stressors: High-stress levels, such as financial difficulties, relationship problems, or significant life events, can contribute to developing PPD. Transitioning to motherhood brings numerous challenges and adjustments, and when combined with external stressors, it can further increase the risk of developing depressive symptoms [18].

Pregnancy or birth complications: Women who have experienced complications during pregnancy or childbirth, such as preterm birth, medical complications, or difficulties with the baby's health, may be more susceptible to PPD. These challenges can heighten stress levels, disrupt maternal-infant bonding, and create additional emotional and physical burdens, increasing the risk of depressive symptoms [19].

Lack of sleep: Sleep deprivation is a prevalent experience during the early postpartum period, primarily due to the demands of infant care, nighttime feedings, and adjustment to a new sleep schedule. It is essential to recognize that inadequate sleep can contribute to mood disturbances and increase the likelihood of developing PPD. This is because a complex interplay exists between hormonal changes, altered neurotransmitter functioning, and sleep disruption, collectively creating a vulnerable state for the onset of depressive symptoms. By identifying and comprehending these risk factors, healthcare providers can improve their ability to identify women at higher risk of developing PPD. This knowledge, in turn, can guide screening efforts and facilitate the implementation of appropriate interventions to support women during this vulnerable period [20].

Impact of PPD on maternal care

Changes in Maternal Behaviors and Bonding

PPD can profoundly impact maternal behaviors and the bonding between a mother and her infant. Mothers experiencing PPD often undergo significant changes in their caregiving behaviors, which can disrupt the establishment of a secure and nurturing mother-infant relationship [21]. One noticeable change in maternal behaviors is a reduced responsiveness to the baby's cues. Mothers with PPD may find it challenging to recognize and appropriately respond to their infant's needs, leading to delays or inconsistencies in meeting their baby's needs for comfort, feeding, and interaction. This decreased responsiveness can contribute to a disconnect between the mother and infant, potentially affecting the infant's emotional and social development [22].

Another change that may occur is a decrease in affectionate touch. Mothers experiencing PPD may exhibit less physical contact with their infants, such as cuddling, hugging, and gentle caressing. Affectionate touch is crucial in promoting the emotional connection and bonding between a mother and her baby. The absence or reduction of such touch due to PPD can hinder the formation of a secure attachment, which is vital for the infant's emotional well-being [23]. Mothers with PPD often experience guilt, inadequacy, and self-doubt regarding their parenting abilities. These negative self-perceptions can undermine their confidence in their caregiving skills and abilities. They may constantly question themselves, fearing they are not good enough mothers or failing to meet their infants' needs. These feelings of inadequacy can create a barrier to engaging in sensitive and responsive caregiving practices, as mothers may doubt their ability to nurture and care for their infants appropriately [24].

Reduced responsiveness, decreased affectionate touch, and negative self-perceptions can significantly impact mother-infant bonding. Infants thrive on consistent, sensitive, and responsive interactions with their caregivers, promoting security, trust, and emotional well-being. When these elements are disrupted due to PPD, establishing a secure and nurturing mother-infant relationship may be compromised [25].

Effects on Maternal-Infant Attachment

PPD significantly impacts the development of maternal-infant attachment, a fundamental element in an infant's emotional and social growth. Maternal-infant attachment is the strong emotional bond and connection between a mother and her baby. It provides a secure base from which the infant explores the world and regulates their emotions [26]. However, mothers experiencing PPD often face difficulty establishing a secure and robust emotional bond with their infants. These challenges can manifest as reduced emotional warmth, characterized by a lack of affectionate touch, limited eye contact, and diminished expressions of joy or delight towards their baby. The diminished emotional warmth may stem from the mother's emotional struggles, such as sadness, fatigue, or anxiety associated with PPD [27]. Understanding the complexities of maternal-infant attachment and its disruption due to PPD is crucial for developing targeted interventions to support mothers and foster healthy infant development.

Inconsistent responsiveness is another characteristic observed in mothers with PPD. They may have difficulty consistently attending to their baby's needs and responding promptly and appropriately to their cues. This inconsistent responsiveness can lead to confusion and distress in the infant, as they may struggle to understand and predict their caregiver's behaviors and responses [28]. Mothers with PPD may also experience difficulties attuning to their baby's emotional signals. Attunement involves the mother's ability to understand and interpret her baby's emotions and respond sensitively. However, due to the emotional turmoil associated with PPD, mothers may find it challenging to accurately interpret their infant's cues and respond with appropriate sensitivity and responsiveness [29].

These difficulties in maternal-infant attachment can have long-term implications for the infant's socio-emotional development. Insecure attachment patterns may emerge, which are characterized by ambivalence, avoidance, or resistance in the infant's interactions with their mother. These insecure attachment patterns can result in emotional and behavioral problems, such as difficulties regulating emotions, reduced self-confidence, and impaired social relationships as the child ages [30]. It is essential to recognize the effects of PPD on maternal-infant attachment and provide appropriate support and interventions to promote healthy attachment relationships. Early detection, intervention, and targeted therapies can help mothers with PPD develop more secure and nurturing relationships with their infants, fostering positive socio-emotional development and well-being for both the mother and child [31].

Implications for Breastfeeding and Infant Feeding Practices

Breastfeeding promotes infant health and development and facilitates the mother-infant bond. However, PPD can challenge successful breastfeeding and infant feeding practices. Mothers with PPD may experience difficulties initiating and maintaining breastfeeding due to decreased motivation, low energy levels, and negative emotions impacting their commitment to breastfeeding [32]. Additionally, certain antidepressant medications for PPD treatment may affect breastfeeding. Some medications can pass into breast milk, potentially affecting infant health. This can create a dilemma for mothers with PPD who must weigh the benefits of treatment against potential risks to their infants [33].

These various impacts of PPD on maternal care have significant consequences for mothers and infants. Understanding these effects is crucial for healthcare providers to provide appropriate support and interventions to mitigate the negative outcomes and promote the well-being of both mothers and infants during the postpartum period [34].

Consequences for infants

Emotional and Cognitive Development

PPD can profoundly affect infants' emotional and cognitive development. Infants rely heavily on their mothers' emotional availability and responsiveness to develop secure attachments and regulate their emotions. However, when mothers experience PPD, they may struggle to provide the consistent and emotionally attuned caregiving that infants need [27]. Infants of mothers with PPD may be exposed to a less responsive caregiving environment, where their cues and signals may go unrecognized or unmet. This lack of responsiveness can lead to difficulties in emotional regulation and developing secure attachments. Infants may experience higher levels of distress and emotional dysregulation, manifesting as increased fussiness, difficulty self-soothing, and more frequent and intense emotional reactions [8,10,11].

Cognitively, infants of mothers with PPD may face challenges in their cognitive development. The reduced engagement and interaction with their depressed mothers can limit their exposure to stimulating and enriching experiences crucial for optimal cognitive development. Infants may have limited opportunities for language-rich interactions, which can result in delays in language development. Similarly, their cognitive functioning and overall intellectual abilities may be impacted as they miss the cognitive stimulation and learning opportunities from responsive interactions with their caregivers [35]. The effects on emotional and cognitive development are interconnected. Emotional well-being and secure attachments provide a foundation for cognitive development, as positive emotional experiences and secure attachments create an optimal environment for infants to explore, learn, and develop cognitive skills [36].

It is important to recognize and address the impact of PPD on infants' emotional and cognitive development. Early detection and intervention, along with targeted support and therapy for affected mothers, can help mitigate these effects and promote healthier emotional and cognitive development in infants. By providing resources, education, and interventions that support the emotional well-being of both mothers and infants, we can foster healthier relationships and better outcomes for infants affected by PPD [3,10].

Behavioral Outcomes

Sleep disturbances are another behavioral outcome commonly observed in infants of mothers with PPD. These infants may experience difficulties establishing regular sleep patterns, have frequent nighttime awakenings, and struggle with falling or staying asleep. Sleep disruptions can exacerbate infants' and mothers' challenges, leading to increased fatigue and decreased overall well-being [37,38].

Feeding problems are also prevalent among infants of mothers with PPD. These infants may experience difficulties establishing and maintaining successful breastfeeding, leading to inadequate nutrition and growth. Additionally, they may have challenges accepting different textures and transitioning to solid foods. Feeding difficulties can create additional stress for both mother and infant, further impacting the overall well-being of the dyad [39].

Furthermore, the impact of PPD on behavioral outcomes can extend beyond infancy. Children of mothers with PPD have an increased risk of developing behavior problems later in childhood and adolescence. These behavioral difficulties can manifest in various ways, ranging from attention and hyperactivity issues to internalizing problems such as anxiety and depression. Children may struggle with impulse control, emotional regulation, and social interactions. These behavioral problems can have long-term implications for academic performance, social relationships, and mental health [40].

Long-Term Effects on Infant Health and Well-Being

The long-term effects of PPD on infant health and well-being are significant and extend beyond the early developmental years. Infants of mothers with PPD are at an increased risk of experiencing various physical health problems. The compromised immune function associated with PPD can make these infants more susceptible to illnesses and infections. Slower growth rates and delays in physical development may also be observed in infants affected by PPD [5]. Furthermore, the adverse effects of PPD can persist into childhood and beyond. Children exposed to PPD may encounter difficulties in academic performance, including lower cognitive abilities, decreased school readiness, and impaired learning skills. These challenges can have a lasting impact on their educational attainment and future opportunities [41].

Social relationships may also be affected by PPD in infancy. These children may experience difficulties establishing and maintaining healthy relationships with peers and family members. They may exhibit behavioral problems, such as aggression, social withdrawal, and emotional instability, further hindering their social development [42]. Additionally, the mental health of children exposed to PPD is at risk. They have an increased vulnerability to developing mood disorders, including depression, anxiety disorders, and other psychological problems, as they grow older. Early exposure to a less nurturing and emotionally responsive caregiving environment can contribute to developing these mental health challenges [43,44].

Interventions and support for mothers with PPD

Screening and Early Detection

Screening for PPD is an essential aspect of comprehensive postnatal care and involves the systematic evaluation of new mothers' mental health status to identify those experiencing PPD. Using standardized screening tools during routine postpartum visits enables healthcare providers to promptly recognize and support at-risk or affected mothers [45].

These screening tools ensure consistency and objectivity in the assessment process and are specifically designed to detect symptoms of PPD and assess its severity. Commonly employed screening tools include the Edinburgh Postnatal Depression Scale (EPDS) and the Postpartum Depression Screening Scale (PDSS). These tools include questions targeting the mother's mood, anxiety, sleep patterns, and overall well-being [46].

By integrating screening for PPD into routine postpartum visits, healthcare providers can proactively identify at-risk mothers and provide timely intervention and support. Early detection is crucial because it allows for promptly initiating appropriate interventions, such as counseling, psychotherapy, or pharmacological treatments, if necessary. It also ensures that mothers receive the support they need during a vulnerable period, which can significantly improve outcomes for both mothers and infants [10,43,46].

Identifying PPD early on enables healthcare providers to offer targeted interventions that can alleviate symptoms, improve maternal well-being, and promote the establishment of a positive mother-infant bond. Prompt intervention can also help prevent the escalation of symptoms, reduce the risk of complications, and mitigate the potential long-term consequences for mothers and infants [12,26,29]. In addition to screening, healthcare providers should maintain open and non-judgmental communication with mothers, creating a safe space for them to express their feelings and concerns. Building trust and rapport with mothers encourages them to disclose their emotional struggles, facilitating early detection and appropriate intervention.

Psychotherapeutic Interventions

Psychotherapeutic interventions, such as cognitive-behavioral therapy (CBT) and interpersonal therapy (IPT), have effectively treated PPD. These interventions are designed to address the specific challenges and symptoms experienced by mothers during the postpartum period [47].

CBT is a widely used approach that identifies and modifies negative thought patterns and beliefs that contribute to developing and maintaining PPD. The therapist works collaboratively with the mother to help her recognize and challenge distorted thinking, develop more realistic and positive thoughts, and build healthier coping mechanisms. CBT emphasizes the importance of behavioral activation, encouraging mothers to engage in enjoyable and fulfilling activities that promote positive mood and well-being [48].

IPT is another psychotherapeutic approach commonly used to treat PPD. It focuses on the interpersonal relationships and social support networks of the mother. IPT aims to improve communication skills, address relationship conflicts or role transitions that may contribute to depressive symptoms, and enhance social support. The therapist helps the mother identify and navigate interpersonal challenges, develop effective communication strategies, and build or strengthen supportive relationships [49].

Psychotherapeutic interventions can be delivered individually or in group settings, depending on the preferences and needs of the mother. Individual therapy provides a one-on-one therapeutic relationship that allows for personalized attention and tailored treatment. Group therapy, on the other hand, provides a supportive environment where mothers can share their experiences, receive validation, and learn from one another's perspectives and coping strategies. Group therapy can foster a sense of community and reduce feelings of isolation, allowing mothers to realize that they are not alone in their struggles [50].

In both individual and group settings, psychotherapeutic interventions for PPD provide a safe and confidential space for mothers to express their feelings, receive emotional support, and develop effective strategies for managing their symptoms. These interventions not only target the immediate symptoms of PPD but also aim to enhance the mother's overall well-being and quality of life [51].

Pharmacological Treatments

Pharmacological treatments are important in managing PPD when other interventions, such as psychotherapy, may not be sufficient or feasible. One commonly prescribed class of antidepressant medications for PPD is selective serotonin reuptake inhibitors (SSRIs). SSRIs work by increasing the availability of serotonin, a neurotransmitter that regulates mood [13].

When determining the appropriateness of pharmacological treatment for PPD, healthcare providers carefully evaluate the potential risks and benefits. They take into account the individual circumstances of the mother, including the severity of symptoms, the impact on daily functioning, and the presence of any comorbid conditions. Additionally, healthcare providers consider the mother's breastfeeding status, as some medications can pass into breast milk [52].

Collaboration between healthcare providers and mothers is vital in making informed decisions regarding medication use during the postpartum period. Open and honest discussions should take place to ensure that the mother fully understands the potential benefits and risks associated with pharmacological treatment. Factors such as the potential impact on breastfeeding, possible side effects, and alternative treatment options should be thoroughly discussed. Shared decision-making empowers the mother to actively participate in her treatment plan and make choices that align with her needs and circumstances [53].

Supportive resources and programs

Support Groups

Peer-led support groups provide an invaluable platform for mothers to connect with others who have experienced or are currently facing PPD. These groups offer a safe and non-judgmental space where mothers can share their experiences, express their feelings, and receive emotional support. Connecting with others who empathize with their struggles can help alleviate feelings of isolation and provide a sense of belonging. Support groups may also incorporate educational components, guest speakers, and therapeutic activities to enhance coping skills and resilience [54].

Education and Psychoeducation

Providing mothers with accurate and comprehensive information about PPD is essential for raising awareness and promoting early intervention. Healthcare providers, community organizations, and online resources can offer educational materials explaining the symptoms, risk factors, and treatment options for PPD. Psychoeducation can empower mothers by helping them understand the biological, psychological, and social factors contributing to their condition. By increasing their knowledge about PPD, mothers can recognize their symptoms, seek help sooner, and make informed decisions about their mental health [55].

Home Visiting Programs

Home visiting programs are designed to provide personalized support and guidance to mothers in the comfort of their own homes. Trained professionals, such as nurses or social workers, can visit mothers regularly postpartum to provide education, emotional support, and practical assistance. These professionals can offer guidance on infant care, feeding practices, and self-care strategies. Home visiting programs also allow professionals to assess the mother's well-being, identify signs of PPD, and connect them to appropriate resources for further evaluation and treatment. By delivering support directly to the mother's doorstep, home visiting programs can overcome barriers such as transportation challenges and allow for tailored interventions based on individual needs [56].

Online Resources and Telehealth Services

The digital landscape provides accessible and convenient avenues for mothers to seek information, support, and professional guidance for managing PPD. Online platforms, websites, and mobile applications can offer comprehensive resources, self-assessment tools, and evidence-based information about PPD. Helplines and crisis hotlines staffed by trained professionals can provide immediate support and guidance to distressed mothers. Telehealth services, including video consultations and remote counseling sessions, enable mothers to connect with mental health professionals from the comfort of their homes, overcoming geographical barriers and ensuring access to specialized care. These online resources and telehealth services broaden the reach of support and intervention options, especially for mothers who may face logistical challenges or limited local resources. By implementing these interventions and support mechanisms, healthcare providers, policymakers, and communities can enhance the well-being of mothers with PPD. It is essential to establish a comprehensive and integrated system of care that addresses the unique needs of mothers and promotes the optimal health and development of both mothers and infants [57].

Implications for healthcare providers and policymakers

Importance of Recognizing and Addressing PPD

Early detection and intervention: Healthcare providers are often the first point of contact for postpartum mothers. By receiving adequate training on recognizing the signs and symptoms of PPD, they can identify affected mothers early. Early detection allows for timely intervention, which is crucial in preventing the condition from worsening and improving outcomes for both mothers and infants [58].

Improved maternal well-being: Recognizing and addressing PPD are essential for promoting the overall well-being of mothers. PPD can significantly negatively affect a mother's mental health, functioning, and quality of life. By identifying and addressing the condition, healthcare providers can help alleviate distress, provide support, and guide affected mothers toward appropriate treatment options [59].

Enhanced infant outcomes: Maternal mental health, including PPD, can significantly impact infant outcomes. Infants of mothers with untreated PPD may experience disrupted bonding, reduced stimulation, and compromised emotional regulation. By recognizing and addressing PPD, healthcare providers can contribute to optimizing the development and well-being of infants through early intervention and support [5].

Reduction of long-term consequences: Untreated PPD can have long-lasting consequences for both mothers and infants. It has been associated with adverse outcomes such as impaired mother-infant bonding, disrupted cognitive development in infants, and an increased risk of behavioral and emotional difficulties in children. By recognizing and addressing PPD, healthcare providers can intervene to mitigate these long-term consequences and promote positive outcomes for families [60].

Integration into routine healthcare practices: Recognizing the importance of postpartum mental health and integrating it into routine healthcare practices are crucial. By incorporating screening tools and assessment protocols for PPD into standard postpartum care, healthcare providers can ensure that no mother slips through the cracks. This integration helps create a supportive environment where affected mothers feel comfortable discussing their mental health concerns and seeking appropriate help [61].

Improving Access to Mental Health Services

Improving access to mental health services is crucial in addressing the needs of mothers experiencing PPD. Policymakers and healthcare systems must prioritize this issue to ensure all mothers have timely access to appropriate care and support. Firstly, it is important to integrate mental health services into the standard postpartum care framework. This means ensuring that mental health screenings and assessments are routinely conducted during postpartum check-ups. By incorporating mental health into routine care, healthcare providers can identify symptoms of PPD early on and initiate appropriate interventions promptly [62].

In addition, efforts should be made to reduce barriers that hinder access to mental health services. Cost is a significant barrier for many individuals seeking mental health treatment. Policymakers should work towards improving insurance coverage for mental health services, including coverage for therapy sessions and medications. By reducing the financial burden, more mothers can afford the necessary treatments without hesitation [63].

Transportation can also be a barrier, particularly for women living in rural or underserved areas. To address this issue, policymakers can explore innovative solutions such as telehealth services. Expanding telehealth options allows mothers to access mental health services remotely, eliminating the need for transportation and increasing convenience. This can be especially beneficial for women with limited access to mental health professionals or those facing logistical challenges in attending in-person appointments [64].

Furthermore, reducing the stigma associated with seeking mental health support is crucial. Policymakers should invest in public awareness campaigns to educate the public about PPD and mental health. By promoting understanding and empathy, society can create a supportive environment that encourages mothers to seek help without fear of judgment or discrimination. Another important aspect of improving access is increasing the availability of mental health professionals with expertise in perinatal mental health. Training programs and incentives should be implemented to encourage mental health professionals to specialize in this field. By having more experts who understand mothers' unique challenges and needs during the postpartum period, the quality of care provided to affected women can be significantly enhanced [65,66].

Policy Recommendations for Promoting Maternal Mental Health

Develop comprehensive maternal mental health policies: Governments should prioritize developing and implementing policies that address maternal mental health. This includes establishing screening programs during prenatal and postpartum care to identify women at risk for or experiencing PPD. Early intervention services should be integrated into healthcare systems to provide timely support and treatment for affected mothers. Additionally, support programs tailored to mothers with PPD should be implemented, providing access to counseling, therapy, and support groups to promote recovery and well-being.

Establish supportive workplace policies: Policies should be enacted to create a supportive work environment for mothers during the postpartum period. Adequate maternity leave should allow mothers to recover physically and mentally, bond with their infants, and adjust to their new roles. Flexible work arrangements, such as part-time work, telecommuting options, and phased return to work, should be made available to accommodate the needs of mothers with PPD. Workplace support programs should be implemented to raise awareness about PPD, provide resources for seeking help, and promote a compassionate and understanding atmosphere for affected employees.

Enhance education and awareness: Policymakers can contribute to the well-being of mothers with PPD by supporting initiatives to increase public awareness and understanding of this condition. This can be achieved through educational campaigns targeting the general public, healthcare providers, teachers, and community members. Training programs should be developed to educate healthcare providers on identifying, assessing, and treating PPD, ensuring they can provide appropriate care and support. Similarly, teachers and community members should receive training to recognize signs of PPD and offer appropriate support to affected mothers.

Foster collaboration between healthcare and social service sectors: Policies should encourage collaboration between healthcare providers, social service agencies, and community organizations to ensure a comprehensive and coordinated approach to supporting mothers with PPD. This can involve establishing referral networks between healthcare providers and mental health professionals and strengthening partnerships with community organizations that offer support services for mothers and infants. By working together, these sectors can provide holistic care that addresses mothers' physical, emotional, and social needs and facilitates their recovery from PPD. By recognizing the importance of postpartum mental health, improving access to services, and implementing supportive policies, healthcare providers and policymakers can significantly promote the well-being of mothers and infants affected by PPD. These efforts can lead to healthier families, improved maternal care, and better long-term outcomes for mothers and infants.

## Conclusions

In conclusion, PPD has multifaceted consequences for both mothers and infants. Mothers experiencing PPD may face challenges in providing adequate maternal care, resulting in changes in maternal behaviors, compromised maternal-infant attachment, and difficulties with breastfeeding and infant feeding practices. These consequences can have a long-lasting impact on infants' emotional, cognitive, and behavioral development, as well as their overall health and well-being. Recognizing and understanding these consequences are crucial for healthcare providers, policymakers, and society. While significant progress has been made in understanding PPD and its consequences, further research is needed to enhance our knowledge and inform targeted interventions. Future studies should focus on investigating the underlying mechanisms linking PPD to the observed consequences, identifying effective prevention strategies, and developing tailored interventions that address the unique needs of affected mothers and infants. Additionally, efforts should be made to improve the accessibility and availability of mental health services and support programs for mothers with PPD.
